# Does afatinib plus bevacizumab combination therapy induce positive conversion of T790M in previously-negative patients?

**DOI:** 10.18632/oncotarget.26192

**Published:** 2018-10-05

**Authors:** Akito Hata, Nobuyuki Katakami, Reiko Kaji, Toshihide Yokoyama, Toshihiko Kaneda, Motohiro Tamiya, Takako Inoue, Hiromi Kimura, Yukihiro Yano, Daisuke Tamura, Satoshi Morita, Shunichi Negoro, on behalf of HANSHIN Oncology Group

**Affiliations:** ^1^ Department of Medical Oncology, Kobe City Medical Center General Hospital, Kobe, Japan; ^2^ Department of Respiratory Medicine, Kurashiki Central Hospital, Kurashiki, Japan; ^3^ Department of Thoracic Oncology, Osaka International Cancer Institute, Osaka, Japan; ^4^ Department of Thoracic Oncology, National Hospital Organization Toneyama National Hospital, Toyonaka, Japan; ^5^ Department of Respiratory Medicine, Kobe University Graduate School of Medicine, Kobe, Japan; ^6^ Department of Biomedical Statistics and Bioinformatics, Kyoto University Graduate School of Medicine, Kyoto, Japan; ^7^ Department of Medical Oncology, Takarazuka City Hospital, Takarazuka, Japan

**Keywords:** afatinib, bevacizumab, T790M, rebiopsy, osimertinib

## Abstract

Third-generation epidermal growth factor receptor (EGFR)-tyrosine kinase inhibitors (TKIs) are markedly effective for T790M-positive patients. To confer their clinical benefit to more patients, a novel therapy to induce positive conversion in T790M-negative patients may be possible. We retrospectively reviewed medical records of patients who had received rebiopsy after completion of ABC-study: a prospective phase II study of Afatinib plus Bevacizumab Combination (ABC)-therapy after acquired resistance to EGFR-TKI. Between October 2014 and September 2016, 32 eligible patients were enrolled in ABC-study at our institutes. Eighteen patients were T790M-negative and 14 were T790M-positive before ABC-therapy. Rebiopsy was performed on 13 T790M-negative and 5 T790M-positive patients after progression of ABC-therapy. In 8 (62%) of 13 T790M-negative patients, T790M status changed from negative to positive after ABC-therapy. Seven of these 8 patients underwent osimertinib therapy. The response rate and median time to treatment failure were 86% and 12.2 months, respectively. There were no adverse events ≥grade 3, nor any treatment-related deaths. On the other hand, T790M remained positive after ABC-therapy in all 5 previous T790M-positive patients. ABC-therapy could induce positive conversion of T790M even in previously-negative patients. We hypothesize that ABC-therapy could provoke “clonal selection”, which purifies T790M-positive cancer cells in heterogeneous tumors. Further studies are warranted to confirm this phenomena.

## INTRODUCTION

Epidermal growth factor receptor (EGFR)-tyrosine kinase inhibitor (TKI) is the standard of care for patients with *EGFR*-mutant advanced/metastatic non-small cell lung cancer (NSCLC). The progression-free survival (PFS) is approximately one year, and acquired resistance (AR) is mostly inevitable [1]. T790M point mutation in exon 20 is the major mechanism of AR, accounting for half of all AR mechanisms [2–4]. Third-generation (3G) EGFR-TKIs are markedly effective and safe for T790M-positive patients [5, 6]. Among 3G EGFR-TKIs, osimertinib is the only clinically available agent now. Unfortunately at present, osimertinib is only indicated in T790M-positive patients, thus T790M-negative patients cannot receive clinical benefit from osimertinib. To confer its clinical benefit to more patients, a novel therapy to induce positive conversion in T790M-negative patients may be possible.

We have reported results of our ABC-study evaluating the efficacy and safety of Afatinib plus Bevacizumab Combination (ABC) therapy after AR to EGFR-TKIs [7]. Interestingly, in some patients after completion of this study, T790M status had changed from negative to positive after ABC-therapy. According to this phenomena, we hypothesized that ABC-therapy could induce positive conversion of T790M in previously-negative patients. The aim of this study was to investigate T790M status from rebiopsy results after ABC-therapy in patients who had been enrolled in ABC-study.

## RESULTS

### Patients

We reviewed 32 eligible patients from our ABC-study. Patient characteristics are shown in Table 1. The median age was 66 (range, 48–86) years. Twenty-one (66%) patients were female. The frequencies of sensitive *EGFR* mutation subtypes were 20 (63%) Del-19, 11 (34%) L858R, and 1 (3%) L861Q. The response rate and disease control rate of afatinib plus bevacizumab were 18.8% and 90.7%, respectively. Median PFS was 6.3 months [7]. Eighteen (56%) patients underwent rebiopsy after ABC-therapy. Four patients received chemotherapies between progressive disease (PD) on ABC-therapy and rebiopsy, and remaining 14 patients were rebiopsied without chemotherapies after PD on ABC-therapy. Sensitive *EGFR* mutation status did not change before and after ABC-therapy in all studied cases.

**Table 1:** Patient characterisics

#	Age/Gender	Primary EGFR-TKIs	Sensitive mutation	T790M status before Afa+Bev/Site/Method	Response/PFS (mo)	T790M status after Afa+Bev/Site/Method	Duration/Chemotherapy between PD and rebiopsy
01	79/F	G, E	L861Q	T790M (−)/Lung/Clamp	PR/13.1	/	/
02	56/F	G, E	L858R	T790M (−)/Cardiac effusion/Clamp	SD/5.1	T790M (−)/Cardiac effusion/Clamp	0 mo/None
03	55/F	E	Del-19	T790M (−)/Lung/Clamp	PD/1.4	T790M (+)/Pleural effusion/Clamp	0 mo/None
04	62/F	G, E	L858R	T790M (−)/Lung/Clamp	SD/3.4	T790M (−)/Lung/Clamp	0.4 mo/None
05	63/M	G	Del-19	T790M (−)/Lung/Clamp	PR/9.3	T790M (+)/Lung/Clamp	0.4 mo/None
06	73/M	E	L858R	T790M (−)/Lung/Clamp	SD/8.7	T790M (+)/Lung/Clamp	8.0 mo/CBDCA+PEM
07	59/M	G, E	Del-19	T790M (−)/Pleural effusion/Clamp	PD/1.0	/	/
08	73/M	G, E	L858R	T790M (−)/Lung/Clamp	SD/2.9	/	/
09	82/M	G	Del-19	T790M (+)/Lung/Clamp	SD/10.3	/	/
10	62/M	E	Del-19	T790M (−)/Pleural effusion/Clamp	PR/10.1	T790M (+)/Pleural effusion/MBP-QP	0 mo/None
11	79/F	G	Del-19	T790M (+)/Pleural effusion/Clamp	SD/2.5	T790M (+)/Pleural effusion/MBP-QP	0 mo/None
12	81/F	G, E	Del-19	T790M (+)/Lung/Clamp	SD/9.1	T790M (+)/Lung/MBP-QP	0.4 mo/None
13	81/F	G	Del-19	T790M (−)/Lung/Clamp	SD/6.3	T790M (+)/Lung/MBP-QP	7.4 mo/GEM+Bev, nab-PTX
14	63/F	G, Afa	Del-19	T790M (+)/Lung/Clamp	SD/2.6	/	/
15	65/F	G	Del-19	T790M (+)/Pleural effusion/Clamp	PD/1.1	T790M (+)/Pleural effusion/Clamp	0 mo/None
16	55/M	G	L858R	T790M (+)/Lung/Clamp	SD/3.5	/	/
17	66/M	G	Del-19	T790M (−)/Lung/Clamp	SD/1.5	/	/
18	74/F	G	Del-19	T790M (+)/Lymph node/Clamp	SD/3.9	/	/
19	66/M	G	Del-19	T790M (+)/Pleural effusion/Clamp	SD/7.8	T790M (+)/Pleural effusion/Clamp	0 mo/None
20	80/M	E	Del-19	T790M (+)/Pleural effusion/Clamp	PR/5.8	/	/
21	64/F	G	Del-19	T790M (−)/Lung/Cobas	PR/5.1	T790M (+)/Lung/Clamp	3.4 mo/Afa
22	48/F	G	Del-19	T790M (−)/Lung/MBP-QP	SD/2.9	T790M (−)/Lung/Cobas	0.1 mo/None
23	68/F	G, E, Afa	Del-19	T790M (−)/Lung/MBP-QP	SD/5.5	/	/
24	80/M	E	Del-19	T790M (−)/Lung/MBP-QP	SD/7.3	T790M (−)/Lung/Clamp/MBP-QP	0.3 mo/None
25	86/F	G, E	L858R	T790M (+)/Lung/Clamp	SD/8.1	/	/
27	79/F	G	L858R	T790M (+)/Lung/Clamp	SD/9.3	/	/
28	74/F	G	L858R	T790M (−)/Lung/MBP-QP	SD/4.1	T790M (+)/Lung/MBP-QP	0.2 mo/None
29	46/F	G	Del-19	T790M (+)/Lung/MBP-QP	SD/9.5	T790M (+)/Lung/MBP-QP	0.4 mo/None
30	65/F	E	L858R	T790M (−)/Lung/MBP-QP	SD/3.1+	/	/
31	67/M	Afa	Del-19	T790M (+)/Pleural effusion/Clamp	PR/9.9+	/	/
32	51/F	G, E, Afa	Del-19	T790M (−)/Lung/MBP-QP	SD/7.8	T790M (+)/Lung/MBP-QP	0.4 mo/None
33	62/F	E, Afa	Del-19	T790M (−)/Lung/Cobas	SD/2.8	T790M (−)/Pleural effusion/Clamp	3.3 mo/4 regimens^#^

Abbreviations: M, male; F, female; EGFR-TKI, epidermal growth factor receptor-tyrosine kinase inhibitor; G, gefitinib; E, erlotinib; Afa, afatinib; PR, partial response; SD, stable disease; PD, progressive disease; PFS, progression-free survival; mo, months; CBDCA, carboplatin; PEM, pemetrexed; GEM, gemcitabine; Bev, bevacizumab; nab-PTX, nanoparticle albumin bound-paclitaxel; Clamp, peptide nucleic acid-locked nucleic acid PCR clamp method; and mutation-biased PCR quenching probe method.

Case 26 and 34 were ineligible.

^#^, docetaxel plus ramucirumab, nab-PTX, Gefitinib+GEM, S-1+Bev.

### T790M status before and after afatinib plus bevacizumab

**Figure 1:**
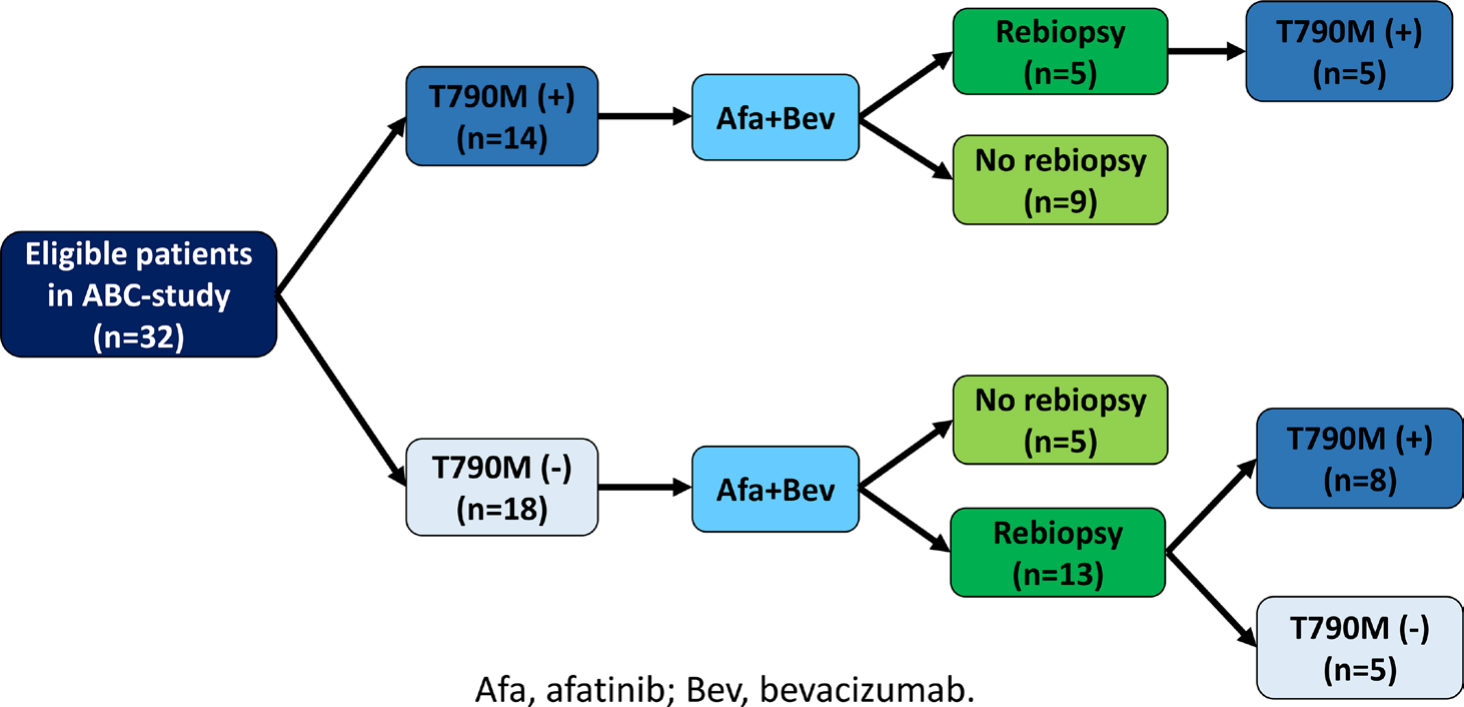
T790M status before and after afatinib plus bevacizumab

Figure 1 shows T790M status before and after ABC-therapy. Eighteen patients were T790M-negative and 14 were T790M-positive before ABC-therapy. Rebiopsy was performed on 13 of 18 T790M-negative and 5 of 14 T790M-positive patients after progression on ABC-therapy. In 8 (62%) of 13 T790M-negative patients, T790M status changed from negative to positive after ABC-therapy. On the other hand, T790M remained positive after ABC-therapy in all 5 previous T790M-positive patients.

### Osimertinib efficacy in cases converted from T790M-negative to -positive after afatinib plus bevacizumab

**Figure 2:**
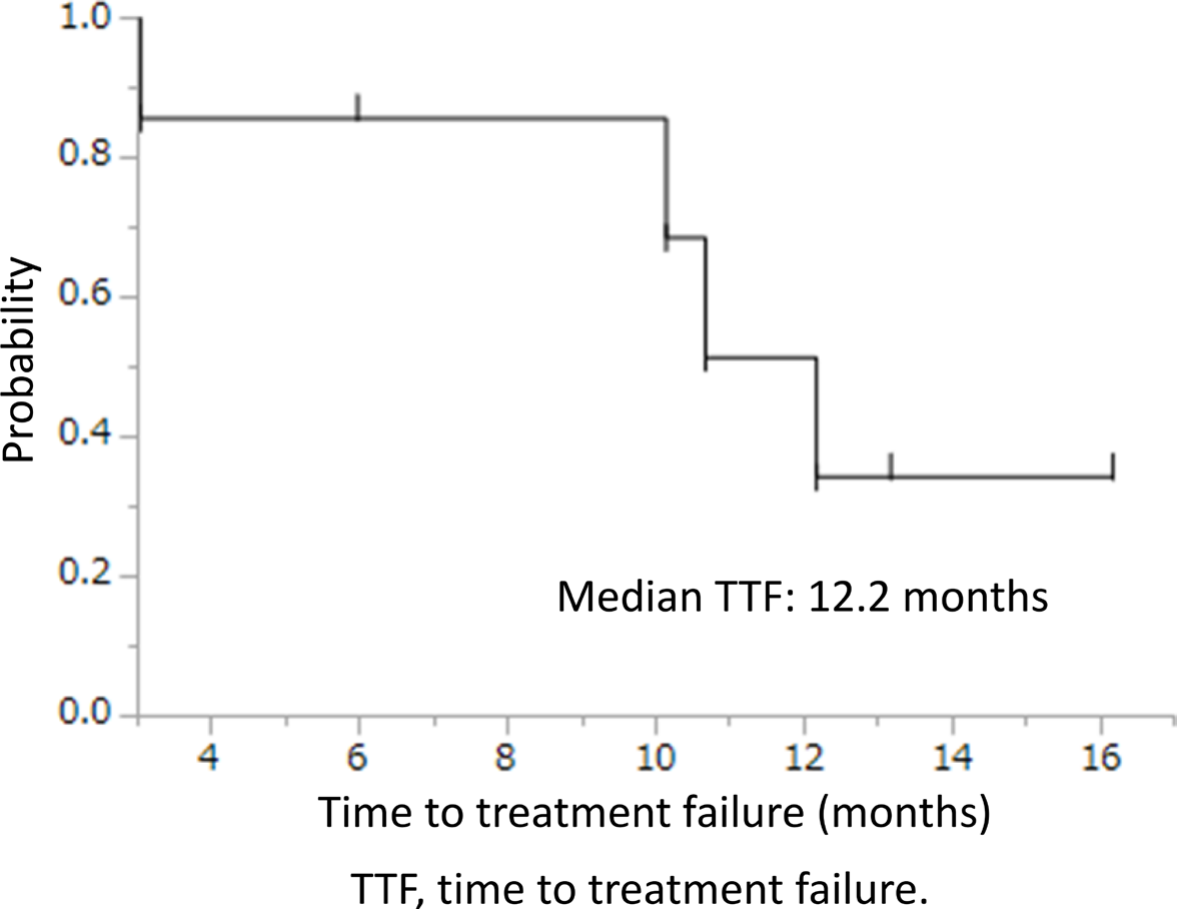
Time to treatment failure of osimertinib

Seven of 8 cases where T790M status changed from negative to positive after ABC-therapy underwent osimertinib therapy. The response rate and disease control rate were 86% and 100%, respectively. The median time to treatment failure and overall survival were 12.2 (95% CI, 3.0-undeterminable) months (Figure 2) and not reached, respectively. There were no adverse events ≥grade 3, nor any treatment-related deaths.

### Case presentation

**Figure 3:**
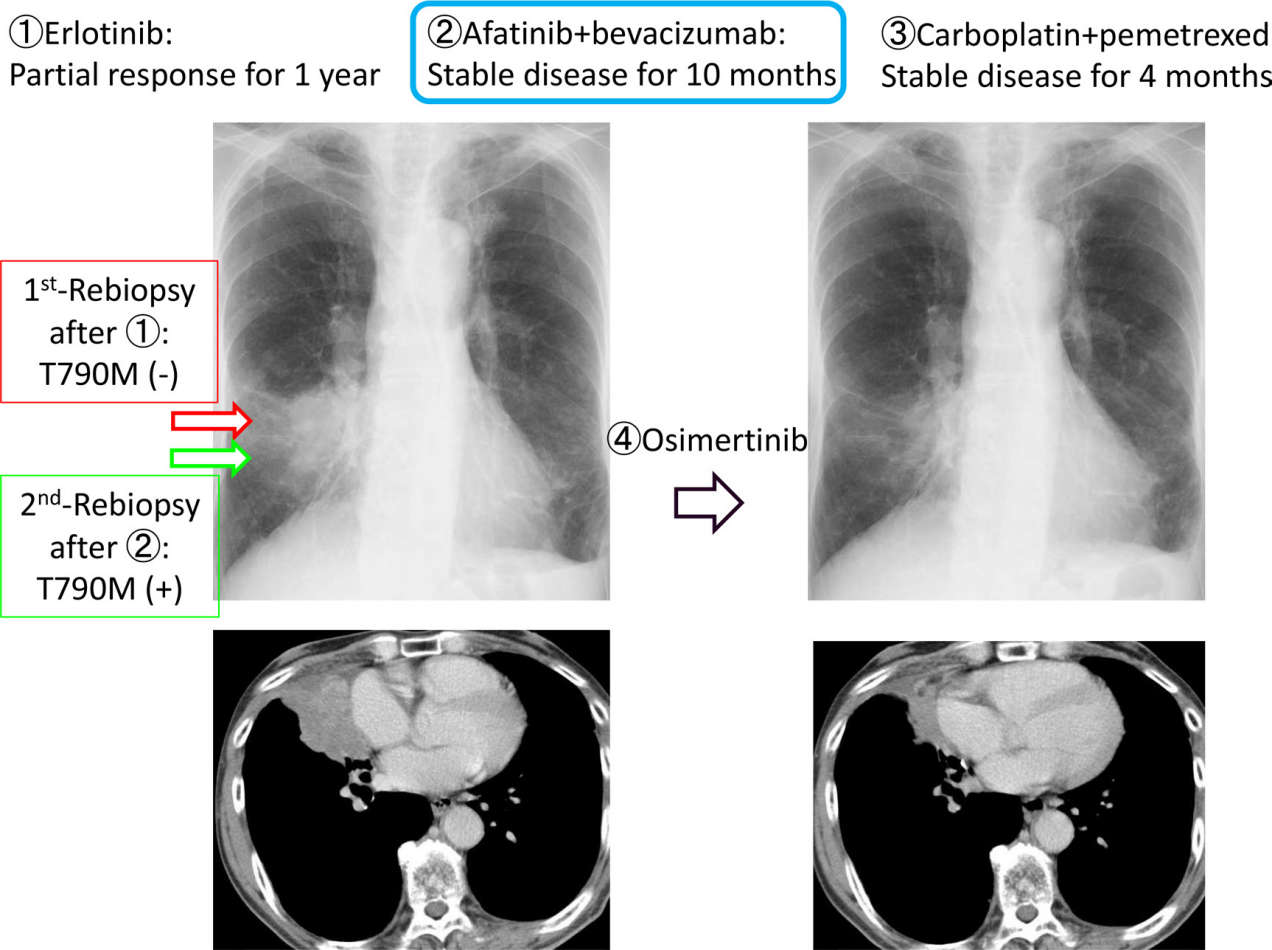
Presentation of case #6

We herein describe a representative case of T790M-positive conversion. The patient was 73 years-old male with *EGFR*-mutant (L858R) NSCLC. He received erlotinib as the first-line therapy for 1 year. After progression, rebiopsy was performed using bronchoscopy. T790M was negative, then ABC-therapy and carboplatin plus pemetrexed were administered as second- and third-line chemotherapies. After further progression, rebiopsy was carried out again to the same lesion and procedure. T790M was converted to positive, and osimertinib was initiated. Partial response was confirmed, and TTF was 12.2 months (Figure 3).

## DISCUSSION

We have observed a positive conversion of T790M in some previously-negative patients with *EGFR*-mutant NSCLC. In 8 (62%) of 13 T790M-negative patients, T790M status changed from negative to positive after ABC-therapy. A few studies found 30–40% of T790M-positive conversion in previously-negative patients by sequential rebiopsy results [8, 9]. Some studies have also shown T790M conversion from negative to positive [10, 11]. Our 62% of T790M-positive conversion is relatively higher than these previous reports, suggesting causes other than false negatives from previous T790M-negative.

**Figure 4:**
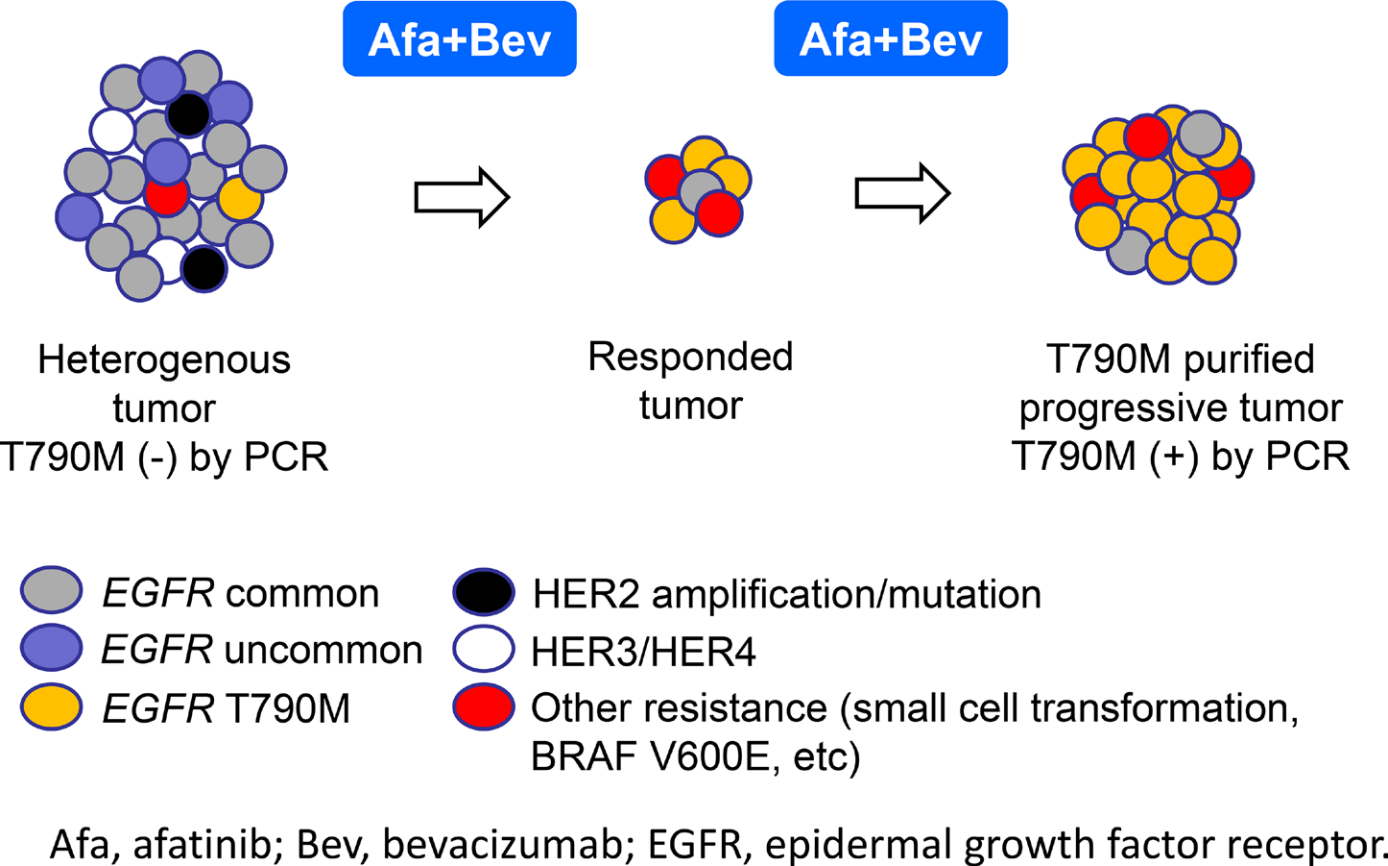
Hypothesis of “clonal selection”

We assumed three mechanisms which caused this phenomenon. First is our hypothesis: clonal selection induced by ABC-therapy. Afatinib plus bevacizumab exerts a strong synergistic effect against cancer cells after acquired resistance to EGFR-TKIs [12]. Afatinib also has high sensitivity to cancer cells harboring uncommon/minor *EGFR* mutations and HER2-4 [13, 14]. Erlotinib plus bevacizumab was reported to be potentially effective against MET-amplified cancer cells after AR in preclinical study [15]. This data might imply similar sensitivity of afatinib plus bevacizumab to MET-amplified cancer cells. ABC-therapy could eliminate heterogenous clones other than T790M-positive clones, and could purify T790M-positive clones in heterogenous tumors (Figure 4). Second, spatiotemporal heterogeneity of T790M could affect this phenomenon. We previously reported T790M spatiotemporal heterogeneity suggested by results of multiple rebiopsy [11]. In patient #3, rebiopsy was done to lung before ABC-therapy, and to pleural effusion after ABC-therapy. This case implies spatial T790M heterogeneity between lung and pleural effusion. Some cases might have exhibited a temporal T790M heterogeneity. Before ABC-therapy, selective pressure from prior EGFR-TKIs might have been insufficient to change T790M-negative results. After ABC-therapy, selective pressure might have increased enough and might have induced T790M-positive results [11]. This T790M temporal heterogeneity is due to TKI selective pressure, which may suggest similar phenomenon to our hypothesis of clonal selection. Third, is a possible differential result by different procedural manner. In patient #21, rebiopsy was performed using cobas before ABC-therapy, and using PNA-LNA PCR clamp after ABC-therapy. Sensitivity of cobas and PNA-LNA PCR clamp are considered as 5% and 1%, respectively [5, 16]. That of MPB-QP is regarded as 1% [17]. In some cases with T790M-positive conversion, PNA-LNA PCR clamp was used before ABC-therapy, and MPB-QP after ABC-therapy. Although sensitivities between them are similar, differential procedual manners might have affected T790M-positivity.

In seven cases where T790M status changed from negative to positive after ABC-therapy, the response rate and disease control rate of osimertinib were 86% and 100%, respectively. The median TTF was 12.2 months. These results were similar or slightly better than historical results of osimertinib for pretreated T790M-positive NSCLC [5, 6]. Our hypothesized clonal selection might have affected these favorable results. T790M-purified cancer after ABC-therapy could have responded well to osimertinib. Notably, Sequist et al. have reported longer TTF of osimertinib after afatinib than after gefitinib [18]. They also hypothesize clonal selection by afatinib affected longer TTF. Results of their and our studies could complement each other and support our hypothesis of clonal selection.

Unfortunately, our study is too small to identify significant predictive factors for T790M-positive conversion. However, T790M-positive conversion seemed to be more evident in those with better response to ABC-therapy. Six (75%) of 8 cases with T790M-positive conversion obtaind PR/SD ≥ 6 months. T790M-positive conversion might be associated with response to ABC-therapy, suggesting a possible deeper clonal selection.

Our study was retrospective and small sample size, including several limitations. After ABC-therapy, not all patients underwent rebiopsy. Patients who were able to receive rebiopsy had targetable lesions and relatively higher tumor burdens than those without targetable lesions. This could be a selection bias. Tumor response and PFS of osimertinib were evaluated using the RECIST, but durations of CT scans depended on doctors in charge. These variable durations also could be a bias of our study. This could largely affect PFS, thus we adopt TTF as an evaluation of therapeutic duration. We hypothesized clonal selection by ABC-therapy, but resistant mechanisms in T790M-negative samples were not investigated, and tumor heterogeneity was not definitively confirmed, but just speculation. After approval of next generation sequencers in the near future, compound mutations could be examined before and after ABC-therapy, which could confirm the hypothesis of clonal selection.

Afatinib can inhibit both uncommon *EGFR* mutations and pan-*HER* signals. If clonal selection is induced by only afatinib monotherapy, the T790M incidence after afatinib would be higher than that after gefitinib or erlotinib. However, Wu SG et al. showed that the T790M incidence after afatinib was 47.6%, similar to gefitinib or erlotinib [19]. Sensitivity to afatinib monotherapy might be insufficient, and additional bevacizumab to afatinib could be necessary to induce clonal selection. However, this basic mechanism is unclear. Actual advantage of and reason to convert T790M by additional bevacizumab are unknown and warrant further investigations.

We also should consider “de novo” T790M mutation as another mechanism of T790M-positive conversion. Hata AN et al. showed acquired T790M mutation can occur via either selection of pre-existing T790M-positive clones or genetic evolution of initially T790M-negative drug-tolerant cells [20]. De novo T790M mutation could provoke positive-conversion of an initial T790M-negative tumor.

In conclusion, ABC-therapy could induce positive conversion of T790M even in previously-negative patients. We hypothesize that ABC-therapy could provoke “clonal selection”, which purifies T790M-positive cancer cells in heterogeneous tumors. This ABC-therapy could confer clinical benefit of osimertinib to more patients without T790M by T790M positive conversion. Further studies are warranted to confirm this phenomena.

## MATERIALS AND METHODS

### Study design

This study was a retrospective study involving patients after completion of ABC-study: Afatinib plus Bevacizumab Combination after AR to EGFR-TKIs in *EGFR*-mutant non-small cell lung cancer, a prospective multicenter, single-arm, open-label phase II trial conducted by the HANSHIN Oncology Group to evaluate the clinical efficacy and safety of ABC-therapy after AR in *EGFR*-mutant NSCLC. ABC-study demanded examination of T790M status before enrollment. Patients of unknown T790M status were ineligible. After primary EGFR-TKI therapy before ABC-therapy, all enrolled patients to ABC-study received rebiopsy. The aims of this study were: first, to examine T790M status before and after ABC-therapy; and second, to evaluate the efficacy of osimertinib in cases converted from T790M-negative to -positive after ABC-therapy. The study was conducted in accordance with the Declaration of Helsinki with the approval of institutional review board.

T790M status was examined using relatively sensitive PCR methods such as the peptide nucleic acid (PNA)-locked nucleic acid (LNA) PCR clamp, the mutation-biased PCR quenching probe method or the cobas [5, 16, 17].

Osimertinib was prescribed in our clinical practice. The efficacy and safety were evaluated according to the Response Evaluation Criteria in Solid Tumors (RECIST) version 1.1 and the National Cancer Institute Common Terminology Criteria for Adverse Events version 4.0, respectively.

Time to treatment failure (TTF) and overall survival (OS) curves and 95% confidence intervals (CIs) were estimated by the Kaplan-Meier method. The statistical analyses were performed using JMP 12 (SAS Institute, Inc., Cary, NC, USA).

## References

[1] Lee CK, Wu YL, Ding PN, Lord SJ, Inoue A, Zhou C, Mitsudomi T, Rosell R, Pavlakis N, Links M, Gebski V, Gralla RJ, Yang JC (2016). Impact of Specific Epidermal Growth Factor Receptor (EGFR) Mutations and Clinical Characteristics on Outcomes After Treatment With EGFR Tyrosine Kinase Inhibitors Versus Chemotherapy in EGFR-Mutant Lung Cancer: A Meta-Analysis. J Clin Oncol.

[2] Pao W, Miller VA, Politi KA, Riely GJ, Somwar R, Zakowski MF, Kris MG, Varmus H (2005). Acquired resistance of lung adenocarcinomas to gefitinib or erlotinib is associated with a second mutation in the EGFR kinase domain. PLoS Med.

[3] Kobayashi S, Boggon TJ, Dayaram T, Jänne PA, Kocher O, Meyerson M, Johnson BE, Eck MJ, Tenen DG, Halmos B (2005). EGFR mutation and resistance of non–small-cell lung cancer to gefitinib. N Engl J Med.

[4] Yu HA, Arcila ME, Rekhtman N, Sima CS, Zakowski MF, Pao W, Kris MG, Miller VA, Ladanyi M, Riely GJ (2013). Analysis of tumor specimens at the time of acquired resistance to EGFR-TKI therapy in 155 patients with EGFR-mutant lung cancers. Clin Cancer Res.

[5] Jänne PA, Yang JC, Kim DW, Planchard D, Ohe Y, Ramalingam SS, Ahn MJ, Kim SW, Su WC, Horn L, Haggstrom D, Felip E, Kim JH (2015). AZD9291 in EGFR inhibitor-resistant non-small-cell lung cancer. N Engl J Med.

[6] Mok TS, Wu YL, Ahn ML, Garassino MC, Kim HR, Ramalingam SS, Shepherd FA, He Y, Akamatsu H, Theelen WS, Lee CK, Sebastian M, Templeton A, AURA3 Investigators (2017). Osimertinib or Platinum-Pemetrexed in EGFR T790M-Positive Lung Cancer. N Engl J Med.

[7] Hata A, Katakami N, Kaji R, Yokoyama T, Kaneda T, Tamiya M, Inoue T, Kimura H, Yano Y, Tamura D, Morita S, Negoro S, HANSHIN Oncology Group (2018). Afatinib Plus Bevacizumab Combination after Acquired Resistance to EGFR-TKIs in EGFR-Mutant Non-Small Cell Lung Cancer: Multicenter Single-Arm Phase II Trial (ABC-Study). Cancer.

[8] Kuiper JL, Heideman DA, Thunnissen E, Paul MA, van Wijk AW, Postmus PE, Smit EF (2014). Incidence of T790M mutation in (sequential) rebiopsies in EGFR-mutated NSCLC-patients. Lung Cancer.

[9] Ichihara E, Hotta K, Kubo T, Higashionna T, Ninomiya K, Ohashi K, Tabata M, Maeda Y, Kiura K (2018). Clinical significance of repeat rebiopsy in detecting the EGFR T790M secondary mutation in patients with non-small cell lung cancer. Oncotarget.

[10] Sequist LV, Waltman BA, Dias-Santagata D, Digumarthy S, Turke AB, Fidias P, Bergethon K, Shaw AT, Gettinger S, Cosper AK, Akhavanfard S, Heist RS, Temel J (2011). Genotypic and histological evolution of lung cancers acquiring resistance to EGFR inhibitors. Sci Transl Med.

[11] Hata A, Katakami N, Yoshioka H, Kaji R, Masago K, Fujita S, Imai Y, Nishiyama A, Ishida T, Nishimura Y, Yatabe Y (2015). Spatiotemporal T790M Heterogeneity in Individual Patients with EGFR-Mutant Non-Small-Cell Lung Cancer after Acquired Resistance to EGFR-TKI. J Thorac Oncol.

[12] Ninomiya T, Takigawa N, Ichihara E, Ochi N, Murakami T, Honda Y, Kubo T, Minami D, Kudo K, Tanimoto M, Kiura K (2013). Afatinib prolongs survival compared with gefitinib in an epidermal growth factor receptor-driven lung cancer model. Mol Cancer Ther.

[13] Kobayashi Y, Mitsudomi T (2016). Not all epidermal growth factor receptor mutations in lung cancer are created equal: Perspectives for individualized treatment strategy. Cancer Sci.

[14] Li D, Ambrogio L, Shimamura T, Kubo S, Takahashi M, Chirieac LR, Padera RF, Shapiro GI, Baum A, Himmelsbach F, Rettig WJ, Meyerson M, Solca F (2008). BIBW2992, an irreversible EGFR/HER2 inhibitor highly effective in preclinical lung cancer models. Oncogene.

[15] Furugaki K, Fukumura J, Iwai T, Yorozu K, Kurasawa M, Yanagisawa M, Moriya Y, Yamamoto K, Suda K, Mizuuchi H, Mitsudomi T, Harada N (2016). Impact of bevacizumab in combination with erlotinib on EGFR-mutated non-small cell lung cancer xenograft models with T790M mutation or MET amplification. Int J Cancer.

[16] Nagai Y, Miyazawa H, Huqun Tanaka T, Udagawa K, Kato M, Fukuyama S, Yokote A, Kobayashi K, Kanazawa M, Hagiwara K (2005). Genetic heterogeneity of the epidermal growth factor receptor in non-small cell lung cancer cell lines revealed by a rapid and sensitive detection system, the peptide nucleic acid-locked nucleic acid PCR clamp. Cancer Res.

[17] Nakamura T, Sueoka-Aragane N, Iwanaga K, Iwanaga K, Sato A, Komiya K, Abe T, Ureshino N, Hayashi S, Hosomi T, Hirai M, Sueoka E, Kimura S (2011). A noninvasive system for monitoring resistance to epidermal growth factor receptor tyrosine kinase inhibitors with plasma DNA. J Thorac Oncol.

[18] Sequist LV, Wu YL, Schuler M, Kato T, Yang JC, Tanaka H, Hida T, Lu S, Park K, Laurie S, Bennouna J, Sibilot DM, Märten A (2017). Subsequent therapies post-afatinib among patients with EGFRmutation-positive NSCLC in LUX-Lung (LL) 3, 6 and 7. Journal of Thoracic Oncology.

[19] Wu SG, Liu YN, Tsai MF, Chang YL, Yu CJ, Yang PC, Yang JC, Wen YF, Shih JY (2016). The mechanism of acquired resistance to irreversible EGFR tyrosine kinase inhibitor-afatinib in lung adenocarcinoma patients. Oncotarget.

[20] Hata AN, Niederst MJ, Archibald HL, Gomez-Caraballo M, Siddiqui FM, Mulvey HE, Maruvka YE, Ji F, Bhang HE, Krishnamurthy Radhakrishna V, Siravegna G, Hu H, Raoof S (2016). Tumor cells can follow distinct evolutionary paths to become resistant to epidermal growth factor receptor inhibition. Nat Med.

